# Retraction Note: Strontium doped bioglass incorporated hydrogel-based scaffold for amplified bone tissue regeneration

**DOI:** 10.1038/s41598-024-76673-7

**Published:** 2024-10-22

**Authors:** Hamed Manoochehri, Masoud Ghorbani, Mehrdad Moosazadeh Moghaddam, Mohammad Reza Nourani, Pooyan Makvandi, Esmaeel Sharifi

**Affiliations:** 1 Student Research Committee, Baqiyatallah University of Medical Sciences, Tehran, Iran; 2 Research Center for Molecular Medicine, Hamadan University of Medical Sciences, Hamadan, Iran; 3 Applied Biotechnology Research Center, Baqiyatallah University of Medical Sciences, Tehran, Iran; 4 Center for Materials Interfaces, Istituto Italiano di Tecnologia, 56025 Pontedera, Pisa, Italy; 5 Department of Tissue Engineering and Biomaterials, School of Advanced Medical Sciences and Technologies, Hamadan University of Medical Sciences, Hamadan, Iran

Retraction of: *Scientific Reports *10.1038/s41598-022-14329-0, published online 17 June 2022

The Editors have retracted this Article.

After publication of this Article, concerns about the data presented were brought to the attention of the Editors. Specifically:

• The image panels for Figures 3e and 3g appear to overlap, though they are described as depicting different experimental conditions;

• In Figure 7, several image panels appear to show the same sample, though they are described as depicting different samples or different timepoints.

The Authors provided raw data files for both figures upon request by the Editors. However, further investigation revealed additional image panels in Figure 7 that appear to contain unusual repeating elements within the same image. The Editors no longer have confidence in the reliability of this data or the results presented in this article.

Hamed Manoochehri disagrees with this retraction. Pooyan Makvandi did not explicitly state whether they agree or disagree with this retraction. The remaining Authors did not respond to correspondence from the Publisher regarding this retraction.

